# The third Iranian and the second joint French-Iranian neuromuscular meeting

**Published:** 2017-04-04

**Authors:** Farnaz Sinaei, Farzad Fatehi

**Affiliations:** Department of Neurology, Shariati Hospital, Tehran University of Medical Sciences, Tehran, Iran

**Keywords:** Iran, Neuromuscular, Myopathies, Congresses

Neuromuscular disorders constitute a significant proportion of neurologic complaints. However, it has not been very long since they came into focus in Iran. The first specialized meeting on muscle and nerve disorders was held in Isfahan, 2010. The second gathering took place soon after in Tehran 2011 as a joint program with French experts.^   1 ^ Kerman was the third place to contribute to neuromuscular disorders education by holding the first myology winter school in 2015.

December 24^th^ and 25^th^, 2016, Dr. Shahriar Nafissi and Dr. Farzad Fatehi from Iran (Tehran University of Medical Sciences) and Professor Shahram Attarian from France (Aix-Marseille University) coined another educational event in Iranian neuromuscular calendar. The third Iranian and the second joint French-Iranian neuromuscular meeting on myopathies and neuromuscular junction disorders was held in Tehran, Shariati Hospital with the presence of renowned Iranian and French neuromuscular specialists (Figure 1).

A part of this program was supported by a grant from the Campus France in Jundishapur project in Iran, which aims to stablish high-quality scientific cooperation between French and Iranian researchers.

**Figure 1:**
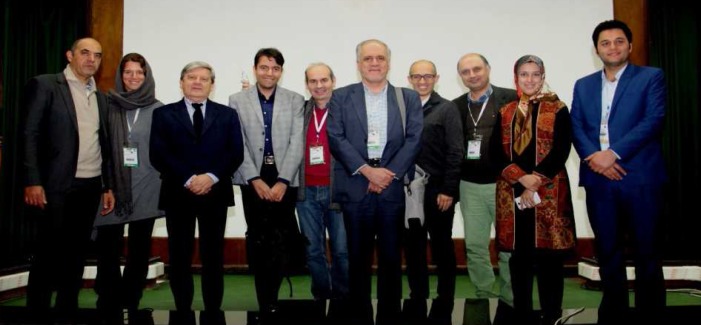
The group of Iranian and French neuromuscular experts

**Figure 2:**
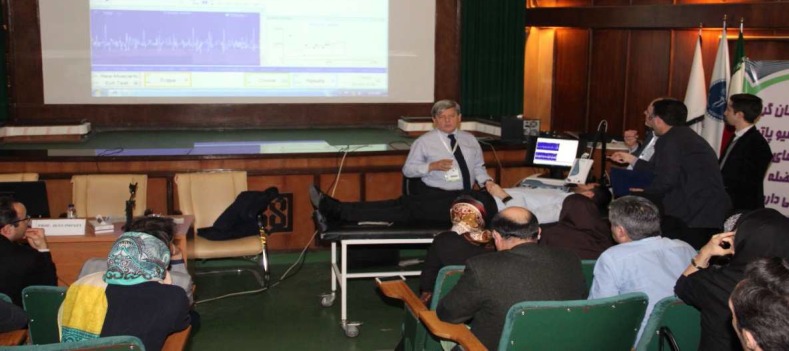
Quantitative electromyography (EMG) workshop by Professor Pouget

Esteemed French guests were Jean Pouget, Shahram Attarian, Jean Mark Leger, Andoni Urtizbera, Emilien Delmont, and Emmanuelle Campana-Salort. About 200 neurologists, physiatrists, and genetists from all over Iran participated in the meeting. The program was divided into two days. The first day was devoted to neuromuscular junction disorders with a particular focus on myasthenia gravis and its novel treatment approaches and also covering congenital myasthenic syndromes (CMS), Lambert-Eaton myasthenic syndrome (LEMS), and Botulism. The second day was designed to address myopathic disorders, and everybody could take advantage of various lectures on inflammatory and metabolic myopathies highlighting the judicious use of diagnostic techniques namely muscle magnetic resonance imaging (MRI), muscle pathology and electromyography (EMG). GNE myopathy was discussed in a separate section as a comprehensive report of Iranian affected patients. Important metabolic myopathies namely Pompe disease, lipid storage, and mitochondrial myopathies were also entailed. Apart from lectures, at the end of each day of the meeting, three challenging neuromuscular patients were introduced and Iranian and French experts discussed them from different perspectives, suggesting additional evaluations and treatments where needed. 

Interactive EMG workshops made the meeting further distinctive. Workshops on single-fiber EMG (by Professor Attarian and Dr. Nafissi) and quantitative EMG (by Professor Pouget) (Figure 2) made everybody stuck in place to the last minutes of the meeting.

The key objectives of the program were to:

- Promote collaborations and joint research programs between Iranian and French neuromuscular experts.
- Give a thorough and concise report of myopathies and neuromuscular junction disorders in Iran.
- Share updates on different aspects of myopathies and neuromuscular junction disorders.

## Notes:

**How to cite this article:** Sinaei F, Fatehi F. The third Iranian and the second joint French-Iranian neuromuscular meeting. Iran J Neurol 2017; 16(2): 105-6.
